# Diabetic Neuropathy: Current Status and Future Prospects

**DOI:** 10.1155/2017/5825971

**Published:** 2017-07-09

**Authors:** Mitra Tavakoli, Dilek Gogas Yavuz, Abd A. Tahrani, Dinesh Selvarajah, Frank L. Bowling, Hassan Fadavi

**Affiliations:** ^1^University of Exeter, Exeter, UK; ^2^University of Manchester, Manchester, UK; ^3^University of Marmara, Istanbul, Turkey; ^4^University of Birmingham, Birmingham, UK; ^5^University of Sheffield, Sheffield, UK

Welcome to this special issue of the Journal of Diabetes research that is focussed on diabetic neuropathy (DN) and features a wide range of articles covering accepted topics related to the epidemiology, pathogenesis, and treatment of different aspects of DN. Diabetic peripheral neuropathy (DPN) and diabetic autonomic neuropathy (DAN) are the most common diabetes-related microvascular complications and can result in significant increase in morbidity, such as chronic pain, foot ulcerations and amputations, and mortality. But despite these significant consequences, current effective screening and treatment strategies are lacking unlike other diabetes-related microvascular complications such as retinopathy and nephropathy. This usually results in delay in the diagnosis of DN till it is well established and more difficult to treat while retinopathy and nephropathy can be detected early using current screening strategies such as retinal images and urinary assessments which allow early interventions to prevent the progression of the disease.

Glucose control is still the only main disease-modifying therapy for diabetic neuropathy, and several disease modification clinical trials for diabetic neuropathy have failed due to lack of sensitive biomarker. There exists an urgent need to identify the most accurate early biomarker of nerve damage to better diagnose DPN in the clinical care of patients and, in particular, to permit an accurate evaluation of future therapies in clinical trials.

This special issue aimed to provide a platform for advance in basic and clinical science in the field of DN.

In an elegant and well-constructed review, L. M. Román-Pintos et al. summarised the literature regarding the epidemiology, risk factors, pathophysiology, diagnosis, and treatments of DN. The review provides important insights into the mechanisms underlying the pathogenesis of DN particularly in relation to oxidative stress, inflammation, and mitochondrial dysfunction providing the experimental basis for each mechanism followed by its translational findings in patients.

N. A. Gavan et al. reported the outcome of the most recent epidemiological study performed in Romania. The study revealed a high prevalence of undisclosed DN, as well as a high prevalence of foot ulcers and amputations in the study population.

A paper by A. A. Tahrani et al. showed ethnic differences in microvascular function in the lower limbs in the South Asian patients with type 2 diabetes compared to White Europeans. In this interesting study, skin microvascular blood flow assessment demonstrated reduced heating flux but preserved acetylcholine response in South Asians. This might be related to the lower prevalence of DPN in South Asians [1].

Patients with diabetes have been reported to have a greater decline in cognitive function and a higher risk of developing dementia. In an interesting study, C.-W. Chang et al. showed in their large study population from Taiwan that regular uptake dosage of aspirin might decrease the risk of developing Alzheimer's disease in patients with type 2 diabetes.

M. C. Perez-Matos et al. reviewed the evidences about lipid-modifying therapies in DPN. The authors concluded that the future research should concentrate on targeting lipids with one or more aggressive interventions specifically in patients whose DPN is detectable but whose progression can still be largely prevented [2].

A. Ando et al. investigated the relationship between macroangiography and DPN applying cardio-ankle vascular index (CAVI) in patients with type 2 diabetes. Their study showed that the CAVI, arterial stiffness, and vascular damage marker have a close relationship with DPN [3].

F. Ishibashi et al. investigated whether the pupillary light reflex (PLR) mediated by intrinsically photosensitive retinal ganglion cells is impaired in type 2 diabetic patients without clinical evidences of autonomic neuropathy. The results showed that blue light induced a more intense and rapid PLR in control subjects and diabetic patients than did red light, and the PLR stimulated by blue light in patients with type 2 diabetes without DAN was more severely impaired than that caused by red light [4].

V. L. Newton et al. demonstrated the increased numbers of neutrophils and levels of L-selectin which is an adhesion molecule important for neutrophil transmigration, in the lumbar spinal cord after 8 weeks of STZ-induced diabetic rats. These findings suggest that dysregulated spinal L-selectin and neutrophil infiltration into the spinal cord could contribute to the pathogenesis of painful DPN [5].

The review by T. Kucera et al. summarised the current view on the etiology, diagnostics, and treatment of Charcot neuropathic osteoarthrophy in diabetes, with particular focus on preserving the extremity through surgical intervention.

Callus formation has long been an important factor to be considered as a predictor for ulceration and subsequent amputation. However, the role of vertical stress (pressure) and shear stress associated with callus has yet to be clarified. A. Amemiya et al. from the Department of Wound Care, Tokyo, Japan, looked into the role of hyperkeratosis (callus) and its link to frictional shear forces and subsequent tissue loss.
